# Mutated Measles Virus Matrix and Fusion Protein Influence Viral Titer In Vitro and Neuro-Invasion in Lewis Rat Brain Slice Cultures

**DOI:** 10.3390/v13040605

**Published:** 2021-04-01

**Authors:** Johannes Busch, Soroth Chey, Michael Sieg, Thomas W. Vahlenkamp, Uwe G. Liebert

**Affiliations:** 1 Institute of Virology, University Hospital Leipzig, Johannisallee 30, 04103 Leipzig, Germany; Soroth.Chey@medizin.uni-leipzig.de (S.C.); liebert@medzin.uni-leipzig.de (U.G.L.); 2 Faculty of Veterinary Medicine, Institute of Virology, Leipzig University, An den Tierkliniken 29, 04103 Leipzig, Germany; Michael.Sieg@vetmed.uni-leipzig.de (M.S.); vahlenkamp@vetmed.uni-leipzig.de (T.W.V.)

**Keywords:** measles virus, reverse genetics, mutagenesis, brain slice culture, neurotropism

## Abstract

Measles virus (MV) can cause severe acute diseases as well as long-lasting clinical deteriorations due to viral-induced immunosuppression and neuronal manifestation. How the virus enters the brain and manages to persist in neuronal tissue is not fully understood. Various mutations in the viral genes were found in MV strains isolated from patient brains. In this study, reverse genetics was used to introduce mutations in the fusion, matrix and polymerase genes of MV. The generated virus clones were characterized in cell culture and used to infect rat brain slice cultures. A mutation in the carboxy-terminal domain of the matrix protein (R293Q) promoted the production of progeny virions. This effect was observed in Vero cells irrespective of the expression of the signaling lymphocyte activation molecule (SLAM). Furthermore, a mutation in the fusion protein (I225M) induced syncytia formation on Vero cells in the absence of SLAM and promoted viral spread throughout the rat brain slices. In this study, a solid ex vivo model was established to elucidate the MV mutations contributing to neural manifestation.

## 1. Introduction

Measles virus (MV), a member of the genus morbilliviruses within the family Paramyxoviridae, causes an acute illness characterized by a high fever, a skin rash, coryza and respiratory symptoms [1]. The virus enters its host by infecting alveolar macrophages via the signaling lymphocyte activation molecule (SLAM, CD150) as a cellular entry receptor [2]. Infected cells migrate to lymph nodes and, as a consequence, more SLAM-positive immune cells (predominantly CD150 expression B- and T-cells) become infected, resulting in viremia and ensuing severe leucopenia [3]. Secondary infections, like bacterial pneumonia, can result and the anergic state of the lymphocytes might impair the immune response for several years [4]. By entry via nectin-4, expressed at the basolateral side of airway epithelium cells and apical budding, MV is released into the lung lumen and can spread to susceptible hosts [5,6]. MV vaccine strains can use CD46 as an additional entry receptor [7], while wildtype (wt) MV cannot. Following the primary measles infection, an autoimmune reaction can cause demyelinization of neurons in approximately 0.1% of infected individuals, with a lethality of about 20% [8]. Other neuronal manifestations can occur by invasion of the virus into the central nervous system (CNS). In immunocompromised patients, onset as measles inclusion body encephalitis (MIBE) is possible, while in immunological-competent individuals, subacute sclerosing panencephalitis (SSPE) induces a slowly progressive but finally lethal disease process in up to 1 in 600 patients following primary measles infection [9]. The process how the virus enters the CNS is not yet fully understood but multiple possible routes have been suggested: (i) infected lymphocytes may act as Trojan horses to cross the blood brain barrier [10]; (ii) endothelial cells become infected and progeny virus is released into brain parenchyma [11]; and iii) virus particles infect the olfactory neurons, are transmitted anterograde and invade the CNS, a process shown for the closely related canine distemper virus (CDV) [12]. MV genomes obtained from SSPE brain tissue display a variety of nucleotide and amino acid changes. Mutations preventing the translation of the functional matrix (M) protein are a key component of neuronal manifestation [13]. On the viral surface, a tetramer of hemagglutinin (H) and a trimer of the fusion (F) protein form the fusion machinery. Receptor binding by the H protein induces conformational changes in the F protein, causing viral entry into the host cell, as reviewed in [14]. In the brain, neither SLAM nor Nectin-4 is present [15,16]. Point mutations in the viral surface proteins (F and H) are linked to neurotropism [17,18,19,20,21,22,23]. Investigations regarding these mutations revealed that they could induce cell-to-cell fusion in cultured cells despite the presence of a known wt MV receptor [24,25,26]. Interestingly, a single [26] or multiple [20,21,23] mutations in the fusion protein are sufficient for neuronal adaptation when H is unaltered.

Animal models have been widely used to investigate the tropism and the pathogenesis of morbilliviruses [12,20,27,28,29]. Besides the infection of animals, organ explants can be used to get insight into viral tropism by mimicking the cytoarchitecture to the in vivo situation [30]. The brain slice culture model [31] provides an alternative to animal experiments and has been used to trace MV spread via live cell imaging [32]. Hippocampal explants of transgenic SLAM-expressing mice were used to investigate the cellular proteins required for viral replication [33] and the role of interferon on neuronal infection [34]. The green fluorescence protein (GFP) expressing wildtype MV-IC323 [35], used in the reverse genetic system in combination with brain slice cultures, represents a powerful tool to investigate the impact of genotypic alterations on viral tropism.

For the present experiments, two MV-IC323 isolates were used that were obtained from three-day-old Lewis rats infected intracranially with MV-IC323. Nucleic acid sequencing was performed of the M, F, H and polymerase (L) genes. In one isolate, a point mutation in the M (A3795G, T120A) as well as in the F gene (A6132G, I225M) was detected compared to the published MV IC-B sequence (Accession no.: AB016162.1). In another isolate, a different point mutation (G4315A, R293Q) in the M and four mutations in the viral polymerase gene (A11116G, E628G; A12693G, I1154V; A13710G, I1493V; and G14406A, V1725I) were detected (Chey, Liebert, unpublished data). In the present study, the biologic significance of point mutations with respect to viral fitness in cultured cells and neural adaptation in the absence of a known wildtype MV receptor was investigated. Using this approach, we aim at contributing to the understanding of viral neural-adaptation.

## 2. Materials and Methods

### 2.1. Mutagenesis

To induce the point mutations detected in the isolates (see Supplementary Materials Table S3) into a wildtype MV background, the pT(+)MV323-eGFP plasmid was used. This plasmid was generated on the basis of p(+)MV323-eGFP [35] (kind gift of Yusuke Yanagi), with alterations as reported previously [36]; i.e., the induction of the cytomegalovirus promoter, the optimization of the T7 promoter by inserting three guanines changed to culture medium containing residues and the insertion of the hammerhead ribozyme. Point mutations were induced using mutagenesis primers (see Supplementary Materials Table S1) and the Primes STAR GXL DNA Polymerase (Takara Bio, Mountain View, CA, USA). Amplification was performed according to the manufacturer’s instructions with slight alterations. The elongation time was reduced to 8 s/kb and 1 ng of template DNA was used. The In-Fusion® HD cloning system (Takara Bio, Mountain View, CA, USA) was used to ligate the PCR products, which were subsequently transformed into *E. coli* by Stellar ™ (Takara Bio, Mountain View, CA, USA), as indicated by the manufacturer. Bacteria were cultured overnight in the presence of kanamycin (50 µg/mL) (Carl Roth, Karlsruhe, Germany). Plasmids were isolated using the ZymoPURE ™ Plasmid MaxiPrep Kit (Zymo Research, Freiburg i. Br., Germany). Whole plasmid DNA sequence was determined using the primers shown in Supplementary Materials Table S2.

### 2.2. Reverse Genetics

The approach to generate MV from DNA was based on the protocol generated by Radecke et al. (1995) [37], with modifications. In brief, HEK 293-3-46 helper cells (provided by Professor Martin Billeter) were transfected with 5 µg of a full-length MV genome-encoding plasmid, 250 ng of the nucleocapsid-coding plasmid pCIAN01 [38] and 20 ng of the viral polymerase-encoding plasmid pEMC-La [37], using calcium phosphate transfection. Following a 24 h incubation at 37 °C, the cells were heat shocked at 42 °C for 60 min. After a further 24 h in culture, the transfected HEK 293-3-46 cells were overlaid on Vero/hSLAM cells [39]. To propagate recombinant viruses, the MV-induced foci were picked and used to infect the Vero/hSLAM cells.

### 2.3. Cell Culture and Virus Propagation

Vero/hSLAM and HEK 293-3-46 cells were propagated as described previously [37,39], under constant G418 (500 µg/mL) selection (Biochrome, Berlin, Germany). Virus stocks were grown on Vero/hSLAM cells using an MOI of 0.001. Prior to cultivation for two to four days at 37 °C, the virus inoculum was removed and OptiMEM (Gibco, Waltham, MA, USA) was applied to the cells. Following ultradeep freezing at −80 °C and thawing, the debris was removed by centrifugation at 5000× *g*, at 4 °C for 5 min. The supernatant was titrated on Vero/hSLAM cells using a plaque assay. In brief, serial dilutions were applied to the cells, incubated for 60 min at 37 °C, 90% humidity and 5% CO_2_. Prior to further incubation for six days, a medium containing 0.6% low-melt agarose was applied. Readout was performed using virus-induced syncytia and fluorescent foci and titers expressed as focus-forming units per mL (FFU/mL). RNA was isolated from generated virus stocks using the QIAamp Viral RNA Mini Kit (Qiagen, Hilden, Germany). To verify the induced point mutations, reverse transcriptions and amplifications were performed with a SuperScript III One-Step RT-PCR System with Platinum™ Taq DNA Polymerase (Thermo Scientific, Waltham, MA, USA). Sequencing was done by Sanger’s dideoxy termination method (Microsynth Seqlab GmbH, Göttingen, Germany).

### 2.4. Growth Kinetics

Replication characteristics of the generated recombinant MV strains were evaluated on Vero/hSLAM cells and Vero cells. Cells were infected with an MOI of 0.01 and incubated up to five days at 37 °C, 95% humidity and 5% CO_2_. Infectious virus particles were quantified in consecutive 24 h steps on Vero/hSLAM cells, as mentioned above.

### 2.5. Immunofluorescence Staining

Infected cells were fixated using 80% acetone (Carl Roth, Karlsruhe, Germany) (*v/v*) at −20 °C for 10 min. Following rinsing with PBS, unspecific binding was blocked using 5% (*w/v*) bovine serum albumin (Carl Roth, Karlsruhe, Germany) in PBS for 30 min at 37 °C. A primary mouse antibody against the MV-N protein (F227, produced inhouse) was diluted in a 1% blocking solution of 1:100 (*v/v*) and incubated for 60 min at 37 °C. Following thorough washing, the secondary antibody (Goat anti-Mouse IgG (H+L) Highly Cross-Adsorbed Secondary Antibody, Alexa Fluor 546, Thermo Scientific, Waltham, MA, USA) was diluted at 1:1000 (*v/v*) in the blocking solution and incubation was done for 30 min at 37 °C. Nuclei were counterstained using DAPI (Carl Roth, Karlsruhe, Germany).

### 2.6. Brain Slice Culture Preparation, Infection and Monitoring

The experiments were approved by the Animal Care and Use Committee of Leipzig University as well as the local government (T33/14 and T36/14). Animals were purchased from Charles River Laboratories (Sulzfeld, Germany). To prepare the brain slice cultures, two-day-old neonatal Lewis rats were used. Tissue preparation was performed as reported previously [40], with slight alterations. In brief, coronal sections were made using a Leica Vibratome vt1000s (Leica, Wetzlar, Germany). The occipital part of the dissected brains was glued to metal chucks and fixated with agarose blocks (4% *w/v* in HBSS). Brain slices were cut to a 300 µm thickness and immediately applied to Millicell cell culture inserts with a polytetrafluoroethylene (PTFE) Biopore ™ membrane and 0.4 µm pore size (Merck, Darmstadt, Germany), pre-equilibrated with HBSS. Slices were cultured using Neurobasal medium ™-A, containing the B27® supplement and penicillin/streptomycin (Gibco, Waltham, MA, USA). After 24 h in the culture, the medium was changed to a culture medium containing 50% (*v/v*) minimum essential medium (MEM), 25% (*v/v*) heat-inactivated horse serum, 12.5 mM HBSS, 12.5 mM HEPES, 0.5 mM L-Glutamin and penicillin/streptomycin (all purchased from Gibco, Waltham, MA, USA). The culture medium was renewed three times a week and the slices assessed microscopically for viability and attachment to the membrane, as described previously [41]. After two days in the culture, the slices became opaque. Following cultivation, the viable and properly attached to the membrane slices became almost transparent. Only transparent slices were used for further analyses. Initially, the viability was verified by applying 5 µM propidium iodide (PI; Carl Roth, Karlsruhe, Germany) to the culture medium and incubation for 2 h. Following incubation, signals were detected using a fluorescence microscope (DMRA–Leica, Darmstadt, Germany). The transparent slices were PI-negative and considered viable.

After 12 days in culture, using each recombinant MV clone, six transparent slices were infected by applying 2500 infectious virus particles in 25 µL to the top of the slice culture. Slices were rinsed with HBSS 60 min later. The following course of infection was monitored using a fluorescence microscope (DMRA–Leica, Darmstadt, Germany) and evaluated 7, 14, 21 and 28 days after the infection. At these indicated time points, the fluorescent cells were counted. Because the exact counting above approximately 150 positive cells was not possible, an ordinate scale was used for the semi-quantitative analyses: no fluorescent cells per slice resulted in “0”; 1 to 10 fluorescent cells per slice resulted in “1”; 11 to 50 fluorescent cells per slice resulted in “2”; 51 to 100 fluorescent cells per slice resulted in “3”; 101 to 200 fluorescent cells per slice resulted in “4”; 201 to 500 fluorescent cells per slice resulted in “5”; and more than 500 fluorescent cells per slice resulted in “6”. This enabled discrimination if much or very much of the cells were GFP-positive (i.e. between 5 and 6). After 28 days post infection (dpi) slices were rinsed with DMEM (Gibco, Waltham, MA, USA) to quantify extracellular infectious virus particles. Three slices of each infection were minced separately using glass beads and a Magna Lyser (Roche, Basel, Switzerland) to isolate the intracellular infectious virus particles. The resulting supernatants were titrated on Vero/hSLAM cells. The remaining slices were fixated using 4% (*w/v*) formalin buffered in PBS (Carl Roth, Karlsruhe, Germany) and used for immunofluorescence staining.

### 2.7. Immunofluorescence of Brain Slices

Fixated slices were permeabilized with 0.2% Triton X-100 (*v/v*) in PBS (TPBS) (Carl Roth, Karlsruhe, Germany) for 30 min at 22 °C. Subsequent blocking was done with 20% (*v/v*) horse serum (Gibco, Waltham, MA, USA) and 2% (*w/v*) bovine serum albumin (Carl Roth, Karlsruhe, Germany) in TPBS for 60 min at 22 °C. Primary mouse antibody against the MV-N protein (F227, produced inhouse) was diluted in the blocking solution to a 1:50 (*v/v*) solution and the slices were incubated for 48 h at 4 °C under constant agitation. Following thorough washing, the secondary antibody was diluted in the blocking solution and incubation was done for 60 min at 37 °C. Nuclei were counterstained using DAPI (Carl Roth, Karlsruhe, Germany). Stained slices were evaluated using a Leica TCS SP8 DMi8 confocal microscope (Leica, Darmstadt, Germany).

### 2.8. Figure Preparation and Statistical Analysis

Figures were prepared using MS Office 2019 (Microsoft©, Albuquerque, NM, USA). Fluorescence microscopic images of the infected brain slices were stitched to generate overviews using the Image Composite Editor software v2.0.3.0 (Microsoft©, Albuquerque, NM, USA). Slight alterations and overlays of the fluorescence channels were done using ImageJ 1.52 n (National Institutes of Health, Bethesda, MD, USA). Statistical analyses were performed using GraphPad Prism 9.0.0 (GraphPad Software, San Diego, CA, USA). A one-way ANOVA followed by Dunnett´s multiple comparison was performed to determine significant differences compared to the parental MV-IC323. To perform a semiquantitative analysis of the neural tropism, an ordinal scaling was used. The statistical evaluation, however, was performed using a test for metric variables (ANOVA).

## 3. Results

### 3.1. Viral Replication

**Figure 1:**
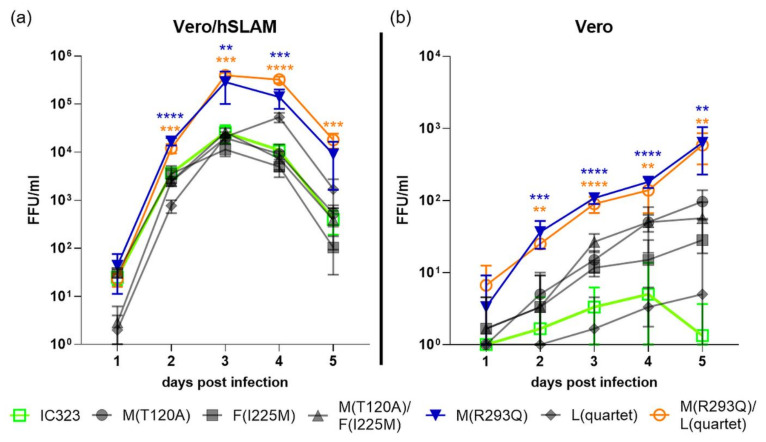
Replication kinetics of the measles virus (MV) clones in the presence (**a**) or absence (**b**) of the wild type MV receptor SLAM. Cells were infected using an MOI of 0.01 and quantification of the infectious virus particles was performed on Vero/hSLAM cells at the indicated time points. Virus clones were designated according to the point mutation induced. Displayed are the mean values of three distinct experiments and the standard deviation. ** = *p* < 0.01; *** = *p* < 0.001; **** = *p* < 0.0001.

To elucidate their effect on the viral replication, the mutations were inserted into the full-length MV genome-encoding plasmid pT(+)MV323-eGFP. According to the isolate they were detected in, the substitutions were induced solely or in the found combination. The whole MV sequence was evaluated in the plasmid to identify unwanted mutations. Only correct plasmids were used to rescue the virus. Generated virus mutant strains were named by the mutations they harbored: MV-M(T120A), MV-F(I225M), MV-M/F(T120A)/(I225M), MV-M(R293Q) and MV-L(E628G/I1154V/I1493V/V1725I), from here on designated MV-L(quartet) and MV-M/L(R293Q)/(quartet). Prior to the experiments, the genome sequence at the sides of the point mutagenesis was determined and only the desired mutations were detected. Replication of the generated clones was compared to the parental virus MV-IC323. All virus clones induced syncytia formation in the Vero/hSLAM cells. To investigate the possible impacts of the induced point mutations on viral replication, infectious viral particles were quantified at various time points. As displayed in Figure 1, a significantly increased replication rate compared to the parental MV-IC323 was induced by the R293Q mutation in the matrix protein. The growth advantages were detected as early as two days post infection and were induced by the M(R293Q) mutation since the L(quartet) mutation solely had no advantageous effect compared to IC323. With the exception of the MV-L(quartet) mutant, which showed 24 h delayed growth, all viruses reached their maximum titers in the Vero/hSLAM cells three days after the infection.

**Figure 2:**
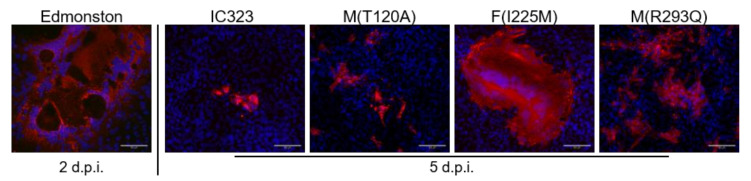
Virus growth in Vero cells lacking the SLAM receptor. Cells were infected using an MOI of 0.01 and the MV nucleocapsid protein (red) as well as nuclei (blue) were stained at the indicated time points. As a positive control for syncytia induction, the MV Edmonston strain was used. Scale bars represent 50 µm. d.p.i. = days post infection.

In Vero cells lacking the SLAM receptor, similar results were obtained. While the parental MV-IC323 replicated at a low level, a significant increase in replication was induced by the M(R293Q) mutation (Figure 1b). This effect could be detected as early as two days after the infection. A trend towards an increase in replication was also detectable due to the M(T120A) and F(I225M) mutation, but this was not significant up to five days post infection. Within the analyzed time period, the N-terminal mutation in the M protein (T120A) caused no significant differences, while the C-terminal R293Q mutation did. To further investigate the effect of the mutations and to elucidate the possibly changed viral properties, immunofluorescence staining in the Vero cells was performed (Figure 2).

To gain insight into the underlying mechanisms of altered viral growth, genetically engineered MV clones and the parental MV-IC323 were compared to the prototype MV Edmonston vaccine strain (MV-Edm) using Vero cells. MV-Edm caused syncytia formation as early as two days after the infection. The MV-IC323 parental and the mutants grew slower and, with the exception of the MV-F(I225M) clone, no cell–cell fusions were induced. The syncytia formation, however, caused no significant increase in produced infectious virus particles, as is shown in Figure 1. The improved replication of the mutated M(R293Q) MV strain compared to the parental MV-IC323 one is not associated with syncytia formation (Figure 2).

### 3.2. Neural Tropism

**Figure 3:**
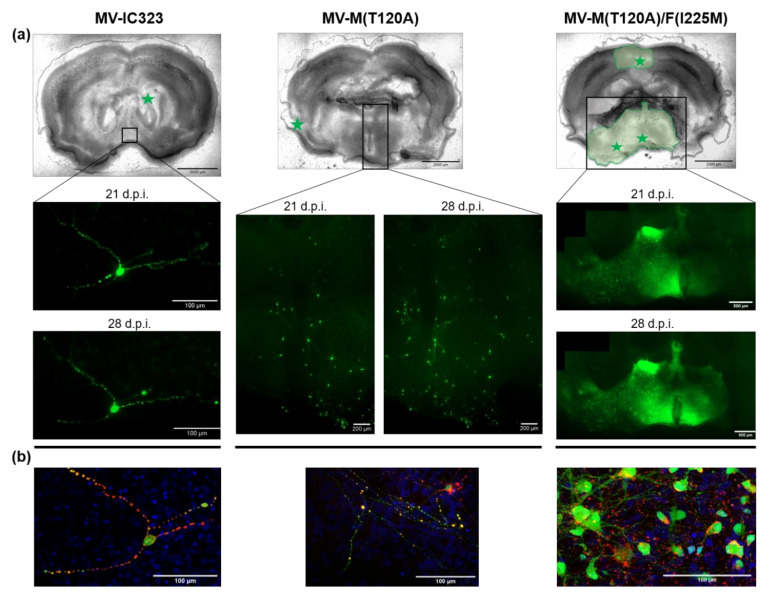
Representative images of virus spread in infected rat brain slice cultures. (**a**) The bright-field microscopy overview of the displayed slices is shown in the top row. Green stars indicate the location of the initially infected cells and green areas illustrate virus spread up to 28 days post infection. Insets highlight the GFP signal as magnifications at the indicated time points. (**b**) Overlay of confocal images taken 28 d.p.i. The MV-N protein is displayed in red, the GFP signal in green and nuclei in blue. d.p.i. = days post infection.

In a further set of experiments, it was investigated whether mechanisms underlying the altered viral replication in Vero cells have an effect upon neural tropism. To elucidate the mutations responsible for possible neuroadaptation, the parental MV-IC323 and the generated viral clones were used to infect the rat brain slice cultures. At seven days post infection, all slices, with the exception of two slices infected with MV-M(T120A)/F(I225M), exhibited infected cells. In the remaining four MV-M(T120A)/F(I225M) infected slices and all six MV-F(I225M) infected slices, the GFP-positive cells increased. In slices infected with the other virus clones and the parental MV-IC323, the number of infected cells decreased during incubation and mainly single cells were positive. The exception was one slice infected with the MV-M(T120A) clone, where GFP-positive cells increased over time but were clearly distinguishable from each other. Representative images of MV-IC323, MV-M(T120A)/F(225M) and one exceptional MV-M(T120A)-infected slice are shown in Figure 3.

**Figure 4:**
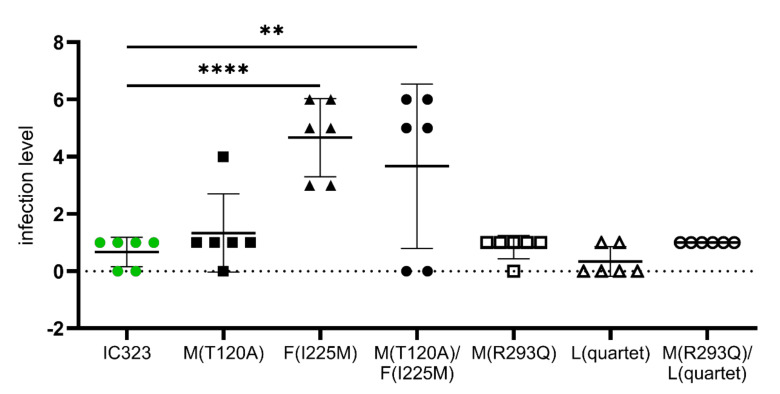
Semi-quantitative assessment of the infection level within every slice 28 days post infection. Displayed are the individual values as well as the mean with the standard deviation, when given. Statistical evaluation has to be considered carefully, since an ANOVA was used to analyze the ordinary-scaled values. ** = *p* < 0.01; **** = *p* < 0.0001.

The visual assessment indicated a neural adaption and hence a promotion of neural spreading due to the F(I225M) mutation. While all clones and the parental virus induced single infected cells within the slices, the mutated fusion protein caused widespread GFP-positive areas. A semi-quantitative categorization of the infection of every slice was performed, whether or not this visual assessment could be verified (Figure 4).

The statistical analysis of the semi-quantitative categorization revealed a significant decrease for GFP-positive cells in slices infected with MV-M(T120A), MV-M(R293Q) and MV-L(quartet). No significant changes were observed in the MV-IC323- and MV-M(R293Q)/L(quartet)-infected slices, also displaying not more than 10 infected cells per slice on average. The M(R293Q) mutation increased the replication in Vero cells but seemed not to contribute to neural adaptation. Slices infected with MV-F(I225M) and MV-M(T120A)/F(I225M), however, showed a significant increase in infected cells from 7 to 14 days post infection, indicating that the infection of neural cells was established. This was further supported by the finding that, in comparison to parental MV-IC323, the F(I225M) mutation bearing MV clones infected significantly more neural cells (Figure 4).

## 4. Discussion

The treatment and prevention of diseases require a broad understanding of their pathological mechanisms. An experimental setup mimicking the in vivo situation is crucial. MV can cause devastating, slowly progressing neuronal malfunction, with no available treatment options and with isolated viral genome sequences displaying various mutations [13,22,23]. To elucidate which mutation might trigger this spread contributes to the understanding of induced pathology. Using reverse genetics, mutations found in MV isolated from rodent brain material were analyzed. A mutation in the carboxy-terminal part of the matrix protein (R293Q) significantly increased the production of infectious virus particles. This effect was detected in the presence as well as the absence of the SLAM receptor in non-polarized Vero cells. Similar effects were reported previously for chimeric MV, where two amino terminal mutations were elucidated as causative [42]. The altered tropism might be caused by the interaction of the M protein with the cytoplasmatic tail of the H protein, since this was reversible by truncation of the tail [43]. Furthermore, a weakened interaction of the M protein with the intracellular F tail has been reported to increase cell-to-cell fusion and reduce infectious virus particle genesis [44]. We did not observe increased syncytia formation or reduced viral particle formation by the M(R293Q) mutation. Thus, the mechanisms, despite an altered interaction with the F tail, might be underlying. For the closely related CDV, it was shown that the cytoplasmic domain of H is dispensable for protein expression and the fusion process but is important for the incorporation into virus-like particles [45]. Since the M(R293Q) mutation increased the production of infectious virus particles in Vero cells with and without SLAM, an effect on particle assembly rather than on receptor usage seems responsible. The amino-terminal mutation M(T120A) had no effect on the viral tropism in unpolarized cells. Furthermore, the mutations in the M protein (R293Q and T120A) did not facilitate replication in the brain slice cultures. In contrast, the F(I225M) mutation caused syncytia formation in Vero cells lacking SLAM and spread in brain slice cultures. Cell-to-cell fusion in the absence of SLAM was also reported for the SSPE-derived F mutation S262G [23] and the tissue culture-derived S262R mutation [21]. These mutations are, like F(I225M), located between the N-terminal and C-terminal heptad repeat region on the F_1_ subunit (HRN and HRC). The S262G mutation allows fusion activation by the H protein without its receptor binding [23]. Single SSPE- or MIBE-derived mutations in the HRC domain (T461I, N462S/N465S and L454W) induce syncytia in Vero cells when expressed with the wt H protein [25,26]. Similar capabilities were induced when six mutations of a SSPE isolate (G168R; E170G; S262G; A440P; R520C and L550P) were introduced simultaneously into a wildtype MV F protein [23]. Moreover, the L454W mutation allows heat-induced fusion activation in the absence of the H protein in vitro [26]. Destabilized phenotypes of F are associated with the neuronal adaptation of MV as well as reduced growth in cultured cells expressing a known receptor [25,46]. We did not observe a disadvantage in the production of infectious virus particles in clones bearing the F(I225M) mutation. Virus propagation at 37 °C had no negative effect on the F(I22M) mutants compared to the parental virus, as reported for L454W and N462K [29]. Even though, virus spread in neural tissue of Lewis rats was caused by the I225M mutation (Figure 4) in the absence of SLAM and Nectin-4. Brain slice cultures were cultivated 12 days prior to the infection to allow astrogliosis to decline and reach a stable culture over a long period [41]. This prolonged cultivation prior to infection might prohibit the interpretation of primary target cells in the CNS in vivo but still renders this approach suitable to determine mutations that promote MV neural adaptation [34]. These findings indicate that the I225M mutation in the wt F protein of MV-IC323 can cause neural adaptation, maybe by a mechanism similar to the one induced by substitution at position S262 [21,23]. Considering no mutations were found in the receptor-binding H protein, this process seems to be driven by F alone and spread in the brain slices might be caused by micro fusion events on the synapsis [47]. The mutations in the large protein had no effect on viral replication. This highlights the conserved structure of this multifunctional polymerase protein within the order Mononegavirales [48].

In the present study, the effect of viral mutations by reverse genetics combined with the slice culture model as an alternative to animal trials was investigated. We have shown that a single mutation in the MV fusion protein (I225M) can render the wt MV-IC323 to spread in brain slice cultures of Lewis rats. Unlike previous studies, no transgenic animals were infected with human-derived MV mutants. In the model presented here, a rodent-derived mutation was analyzed in a rodent model and thus might allow to elucidate the mechanism underlying neuronal adaptation and to draw parallels to human CNS manifestation. The mutation we found in the fusion protein is not located in a yet described functional domain of the F protein and might indicate possible fusion activation in this domain. Further experiments are required to determine the infected cell types and pathology in rats.

## Data Availability

The data set analyzed for the current study is available from the authors upon reasonable request.

## Supplementary Materials

The following are available online at https://www.mdpi.com/article/10.3390/v13040605/s1, Table S1: Primers used to generate MV clones on the basis of pT(+)MV323-eGFP., Table S2: Primers used to sequence the generated MV genome encoding plasmids and viruses; Table S3: Mutations found in the isolates from rat brains.
