# Supplementary material for: Mutated Measles Virus Matrix and Fusion Protein Influence Viral Titer In Vitro and Neuro-Invasion in Lewis Rat Brain Slice Cultures
Source: Viruses. 2021 Apr 1;13(4):605. doi: 10.3390/v13040605 (PMC8066528; doi:10.3390/v13040605)
Supplement: Supplementary file 1 [file viruses-13-00605-s001.pdf]

viruses

Supplementary Material

Table S1: Primers used to generate MV clones on the basis of pT(+)MV323-eGFP. Mutated nucleotides are shown in bolt and underlined. Numbers in the nomenclature indicate the nucleotide position according to the IC-B sequence (accession no.: AB016162.1).

Name	sequence (5´→3´)
MV-M-A3795G-s	CCCCACTA**G**CCCTCCTCACACCTTGGA
MV-M-A3795G-as	GGAGGG**C**TAGTGGGGTGTTGTTGTAGAAC
MV-F-A6132G-s	GACCCCAT**G**TCTGCGGAGATATCCATCCAGG
MV-F-A6132G-as	CGCAGA**C**ATGGGGTCCCGTAAGCTGG
MV-M-G4315A-s	CCTTAATC**A**GTTACTCTGGAGGAGCAG
MV-M-G4315A-as	AGTAAC**T**GATTAAGGTCTTCATTGATATCCATC
MV-L-A11116G-s	TAAAGCAG**G**AAAAGGGTTTGTAGGATTCCCTC
MV-L-A11116G-as	CCTTTT**C**CTGCTTTAACGTTCCTGGTACTTGT
End-MV-L-pTMV-s	CGGATACAGCGCTCTGATTAAGGATTAATTGGTTGAACT
End-MV-L-pTMV-as	AGAGCGCTGTATCCGACTAACTTGTACCATTCTTTGG

Table S2: Primers used to sequence the generated MV genome encoding plasmids.

Name	sequence (5´→3´)
T7-Promotor-as	CCCTATAGTGAGTCGTATT
Leader-MV-N-s	ACCAAACAAAGTTGGGTAAGGATAG
MV-N-s/Leader	ACCAAACAAAGTTGGGTAAGG
N-Start-s	ATGGCCACACTTTTGAGG
MV-N-Q-s	TATYGAAGTGCAAGAYCCTGAGG
Roche-MV-N-s	GGGTCTTGCTCGCAAAGG
MV-N-Q-as	CCTTCTTAGCTCYGAATCAGCTG
Roche-MV-N-as	CCTTCTTAGCTCCGAATCAGC
MV-NLC-s	TTAGGGCAAGAGATGGTGAGG
MV-NLC-as	ATCTCTGAAACAAGCCTTGC
P-vor-Start-as	CGGCTCCAGTCGTGGG
P-Start-s	ATGGCAGAAGAGCAGGC
MV-P1	ACTCCAATCCAGAGGCAACAAC
MV-P2	TTCGGGTGTCCACTCCTGTATC
P-Stop-as	CTACTTCATTATTATCTTCATCAG
P-nach-Stop-s	CTACAGCTCAACTTACCTGC
MV-M-SalI-s	ACCCCATGCCAGTCGAC
MV-M-SalI-as	GTCGACTGGCATGGGGT
MV-M-vor-Start-s	TGATTGCCTCCTAAGTTCCACA
MV-M-Start-s	ATGACAGAGATCTACGATTTC
MV-M-Mitte-s	CCTGCCCTTAGGTGTTGGTAG
MV-M-Mitte-as	CCGTTATCCGAAAGACGGGT
MV-M-vor-ende-s	GGTTTTTGCACTTGGTGGGA
MV-M-vor-ende-as	ATCACGTCGTCGTAAATGCG
F-BstEII-s	GACCGAGGTGACCCAAC
M-BstEII-as	GCGGTTGGGTCACCTC
MV-F-vor-Start-s	CACCGGGAATCCCAGAATCA

**Name**	**sequence (5´→3´)**
MV-F-nach-Start-s	CTGCACGAGGGTAGAGATCG
Roche-MV-F-s	ATCAGGCAATTGAGGCAATC
MV-F-Mitte-s	ATTGGCTGTTCAGGGTGTCC
Roche-MV-F-as	GACACCCTGAACAGCCAATATC
MV-F-Mitte-as	TGTCCCTACGTCCAACCTCT
MV-FLA-fwd	GGTTTATCGAGCACTAGCAT
MV-FLA-rev	GACATACCAACTTGTTCTCC
F-PacI-s	CGGTAGTTAATTAAAACTTAGGGTG
F-PacI-as	CACCCTAAGTTTTAATTAACTACCG
MVH-fwd	CAGATGACAAGTTGCGAATGGA
H-SpeI-s	GCATACCCACTAGTGTGAAATAG
H-SpeI 19nt-s	GATGTCACCCAGACATCAG
H-SpeI-as	CTATTTCACACTAGTGGGTATGC
L-Start-s	ATGGACTCGCTATCTGTCAAC
MV-L 9460-s	CTTAGRAGTTAYCCGGCCCA
MV-L 9742-as	AAACCAAAACAGAAAGGGYTCAA
MV-L 10032-s	TGGTTTCTTCCCTGCACTCG
MV-L 10629-s	GGACAAGGCACTTGCTGCTC
MV-L 10821-as	TTGAACTCAGGGTCATGGAGG
MV-L 11053-s	GGGCCAGTCYYAAAAACCYA
MV-L 11374-as	GGGGCAATGAGGRTCACTYA
MV-L 11670-s	TGAYATTGGCCATCACCTCAA
MV-L 11849-as	CRATATTRCTGCATGCTGCCC
L-NheI-s	CTAGACTGGGCTAGCGAC
L-NheI-as	GTCGCTAGCCCAGTCTAG
MV-L 12571-s	GCAGGGATGGTGCTATTGACA
MV-L 12993-as	TCATCATCACCSKAAGCCCA
MV-L 13167-s	CACAATCTCCAAYGACAATCTCTCA
MV-L 13783-as	YGADGACAACAGCTCACCCA
MV-L 14169-s	GGMAGAGGCTAKGYTATCTCCAGC
MV-L 14406-as	TCATCGTGTGGRGGTCTGAA
MV-L 14962-as	TTTGCCMARGAGYAGAGCCA
MV-L 15114-s	WGAYCTCAARGCTAACCGGC
MV-L 15776-as	TCAGRGCRCTGTATCCGACT
L-Stop-as	TTAATCCTTAATCAGAGCGC
MV-Trailer30-s	ATATATTAAAGAAAACTTTGAAAATACGAA

Table S3: Mutations found in the isolates from rat brains.

Isolate	gene	nucleotide position compared to IC-B (accession no.: AB016162.1)*	amino acid substitution in the protein
1	M	A3795G	T120A
	F	A6132G	I225M
2	M	G4315A	R293Q
	L	A11116G	E628G
		A12693G	I1154V
		A13710G	I1493V
		G14406A	V1725I

**RNA isolation, reverse transcription, amplification and sequencing was performed as described in section 2.3 of the manuscript. Generated sequences were compared to published data using the Basic Local Alignment Search Tool (BLAST; NCBI, USA). Amino acid substitutions were analyzed using GENtle (V1.9.4; University of Cologne, Germany).*
